# Personality similarity predicts synchronous neural responses in fMRI and EEG data

**DOI:** 10.1038/s41598-022-18237-1

**Published:** 2022-08-22

**Authors:** Sandra C. Matz, Ryan Hyon, Elisa C. Baek, Carolyn Parkinson, Moran Cerf

**Affiliations:** 1 Columbia Business School, Columbia University, 3022 Broadway, New York, 10027 USA; 2 UCLA, Los Angeles, USA; 3 Northwestern University, Evanston, USA

**Keywords:** Psychology, Human behaviour, Social neuroscience, Cooperation

## Abstract

Successful communication and cooperation among different members of society depends, in part, on a consistent understanding of the physical and social world. What drives this alignment in perspectives? We present evidence from two neuroimaging studies using functional magnetic resonance imaging (fMRI; *N* = 66 with 2145 dyadic comparisons) and electroencephalography (EEG; *N* = 225 with 25,200 dyadic comparisons) to show that: (1) the extent to which people’s neural responses are synchronized when viewing naturalistic stimuli is related to their personality profiles, and (2) that this effect is stronger than that of similarity in gender, ethnicity and political affiliation. The localization of the fMRI results in combination with the additional eye tracking analyses suggest that the relationship between personality similarity and neural synchrony likely reflects alignment in the interpretation of stimuli and not alignment in overt visual attention. Together, the findings suggest that similarity in psychological dispositions aligns people’s reality via shared interpretations of the external world.

## Introduction

Individuals perceive and experience the same situation, context, item, or concept in their own distinctive way^1^. One person might get excited when encountering a group of people, while another might become anxious and self-conscious. One person might feel inspired by a piece of art, while another might get bored by it and start to mind-wander. What drives the alignment (or lack thereof) in how people perceive and experience the world?

Research at the intersection of neuroscience, psychology and cognitive science has started to explore the roots of neural alignment across three distinct categories: (1) situational drivers, such as shared psychological perspectives, primed interpretive frames or exposure to engaging or emotional content^2–6^, (2) interpersonal drivers, such as close relationships^7,8^, and (3) intrapersonal drivers, such as gender, trait paranoia, cognitive styles or working memory^9–12^. In addition, the existing research distinguishes between neural synchrony as (1) the active process of synchronization between co-present dyads or groups of individuals during social interactions^13,14^, versus (2) the passive synchronization of neural activity across individuals that is evoked by a common stimulus^4,6,15,16^.

Here, we contribute to the literature on intrapersonal drivers of passive synchronization by testing the extent to which neural synchrony is related to people’s enduring psychological characteristics: their personality. Personality traits are thought to capture fundamental individual differences in the way that people think, feel and behave^17^. These traits are known to be relatively stable over time and predictive of a variety of consequential outcomes such as mental health, vocational interests, and relationship quality^18^.

Our study builds on previous research suggesting that personality traits are related to both brain anatomy^19,20^ and function^21–24^. If certain personality traits are reliably related to particular psychological phenomena and their neural underpinnings, we would also expect similarity in personality to be related to similarity in neural responses. However, the translation of prior work to the specific research question we address in this paper—namely how personality similarity is related to similarity in how people perceive and experience the world—is limited by the fact that much of this work focused on either resting-state data (e.g.^25–28^) or neural responses evoked by highly-controlled, task-based paradigms. While these findings provide initial evidence for the neural basis of personality traits, they lack ecological validity^29,30^ and do not directly speak to the impact of *dyadic* personality similarity on neural synchrony. To date, only a single study has investigated the relationship between personality similarity and neural synchrony using a naturalistic fMRI paradigm^11^. The findings provide initial evidence that similarities in individuals’ holistic personality profiles—i.e., similarity across all items in a personality questionnaire—are associated with synchrony in the neural activity evoked by watching naturalistic stimuli.

**Figure 1:**
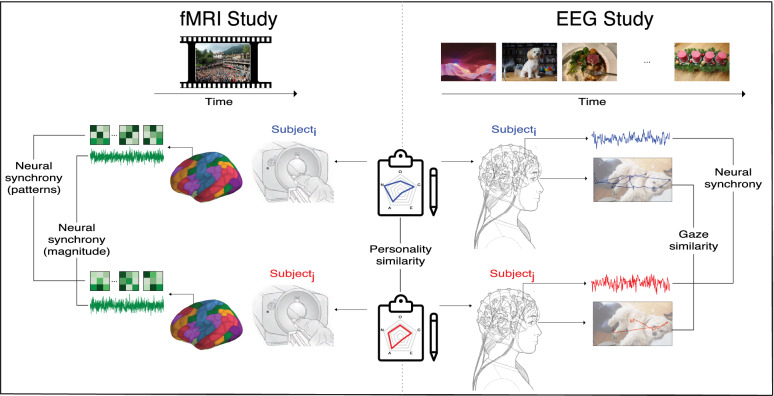
**Experimental paradigms.** Subjects completed personality questionnaires and viewed content while neural data were acquired. Inter-subject personality similarity was calculated using Euclidean distance between subjects’ personality traits (center). In the fMRI study (left), subjects watched a series of videos while being scanned. Temporal fluctuations in spatially-averaged response magnitudes and in spatially-distributed response patterns were extracted from each brain region and subject. These time series were used to compute pattern- and magnitude-based neural synchrony measures for each unique dyad. In the EEG study (right), subjects saw a series of images while data were acquired from their brain. After preprocessing—filtering and artifact removal—the data from pairs of subjects were linearly projected to a transformation that maximizes the correlation between the two individuals in component-space. Averaging the correlation between each pair of components, and, following, across all images viewed, yields a quantitative estimation of the synchrony among the dyad. Simultaneous acquisiton of eye tracking data allowed for parallel comparison of each individual’s scanpath and an estimation of the similarity in viewing trajectories. The images depicted in the figure are different from those shown to participants and were chosen as copyright-free equivalents.

Here, we study the relationship between personality similarity and neural synchrony in two distinct samples: an fMRI sample (Study 1; *N* = 66 with 2145 dyadic comparisons) and an EEG sample (Study 2; *N* = 225 with 25,200 dyadic comparisons; see Fig. 1 for an illustration of the experimental paradigms). In addition to replicating the relationship between personality similarity and neural synchrony^11^, our findings offer three novel contributions. First, we emphasize the unique role of personality similarity in creating neural synchrony by directly comparing the effects of personality to the effects of other socio-demographic characteristics (i.e., age, gender, ethnicity and political ideology). By doing so, we provide a novel take on the scientific and popular discourse around the personal characteristics that separate or connect members of society in their views of the world. Second, we provide granular insights into the specific brain regions and personality facets that drive the relationships between personality similarity and neural synchrony. Third, we shed light on the potential mechanisms underlying the effect of personality similarity on neural synchrony by exploring whether the relationship between the two is driven by similarities in selective attention to certain stimuli (attention-hypothesis) or similarities in interpretation of, or responses to, those stimuli (interpretation-hypothesis). Our conclusions are based both on the investigation of the specific brain regions implicated in the effect (neuroimaging data) as well as eye tracking analyses testing for the mediating role of eye gaze similarity.

Notably, the combination of the fMRI and EEG samples allowed us to test for convergent evidence from two datasets that used different study designs, stimuli, survey measures and neural acquisition methods. The differences in methodologies are partly due the fact that the datasets were collected independently and only combined into a single research project after each data collection had been completed. We highlight the differences in methodology in the respective Methods sections and discuss the resulting limitations and advantages in the General Discussion.

## Study 1

Study 1 investigated the relationship between personality similarity and neural synchrony across different regions of the brain using fMRI. Given the high spatial resolution of fMRI technology, the main goal of Study 1 was to shed light on the specific brain areas in which personality similarity is associated with similar neural responses to the world around us, and to provide initial insights into the cognitive, affective, and perceptual processes that underlie this effect.

The data used in Study 1 were acquired as part of broader collection effort aimed at studying inter-subject correlation and social dynamics^31^.

### Methods

All methods were carried out in accordance with the guidelines and regulations put forward by UCLA. The study protocol in its entirety was approved by UCLA’s Institutional Review Board.

#### Participants and procedure

Seventy subjects participated in the fMRI study and watched a one-hour series of short minutes-long videos spanning a wide range of genres (e.g., comedy, drama, documentary) while undergoing brain scanning. The fMRI session consisted of four consecutive periods. All subjects viewed the stimuli in the same order. After the fMRI session, subjects completed a short personality assessment^32^ and reported their age, gender, and ethnicity.

All subjects were students, fluent in English, and had normal or corrected-to-normal vision. Four subjects were excluded from the analyses (two subjects had excessive movement in more than half of the scans, one subject fell asleep during multiple periods, and one subject ended the scanning session early). We used data from the remaining 66 subjects for analyses (62% female; 18.23 ± 0.63 years old). Of these subjects, one subject had excess movement in only one of the four fMRI runs, and another subject fell asleep during one of the four fMRI periods. Thus, we excluded these scan periods from the analyses involving these two subjects. The subjects identified as Asian (32%), Hispanic/Latinx (29%), Caucasian/White (24%), Mixed (14%), and Black/African (2%). All subjects provided written informed consent.

#### Materials

Stimuli consisted of 14 video clips presented with audio. The durations of the videos ranged from 91 to 734 s (Table S1 in the Supplementary Information). Stimuli were partially adapted from prior work^8^ and were selected such that they were: unfamiliar to most subjects, engaging, and evocative of diverging inferences across viewers. Subjects were told that their experience would be similar to that of watching television while another person “channel surfed”.

#### Measures

##### Personality

We measured subjects’ personality using the Ten-Item-Personality-Inventory (TIPI)^32^. The TIPI is an established short measure of the Five Factor Model of personality^33^ which posits five relatively stable traits: (1) Openness-to-experience, which refers to the extent to which people prefer novelty over convention, (2) Conscientiousness, which captures the extent to which people prefer an organized or a flexible approach to life, (3) Extraversion, which refers to the extent to which people enjoy company and seek excitement and stimulation, (4) Agreeableness, which reflects differences concerning cooperation and social harmony, and (5) Neuroticism, which reflects a tendency to be emotionally volatile or anxious.

With internal consistencies of α = 0.45 for Openness, α = 0.46 for Agreeableness, α = 0.53 for Conscientiousness, α = 0.62 for Neuroticism, and α = 0.75 for Extraversion, the reliability of the measure was found to be comparable to that reported in the scale validation^32^.

##### Neural data

*Data acquisition and preprocessing*

Functional and structural neuroimaging data were acquired using a 3-Tesla Siemens Prisma scanner and preprocessed to correct for instrumental, physiological, and movement-related noise (see Supplementary Information for more details).

*Regions-of-interest parcellation*

We used a cortical parcellation scheme with 200 parcels^34^ for the whole-brain analyses. Each parcel is associated with one of seven cortical networks of the parcellation (dorsal attention, ventral attention, frontoparietal control (FPCN), somatomotor, default mode (DMN), visual and limbic networks; ^35^). In addition we defined 14 subcortical regions using the Harvard–Oxford atlas^36^.

##### Similarity indices

*Neural synchrony*

**Figure 2:**
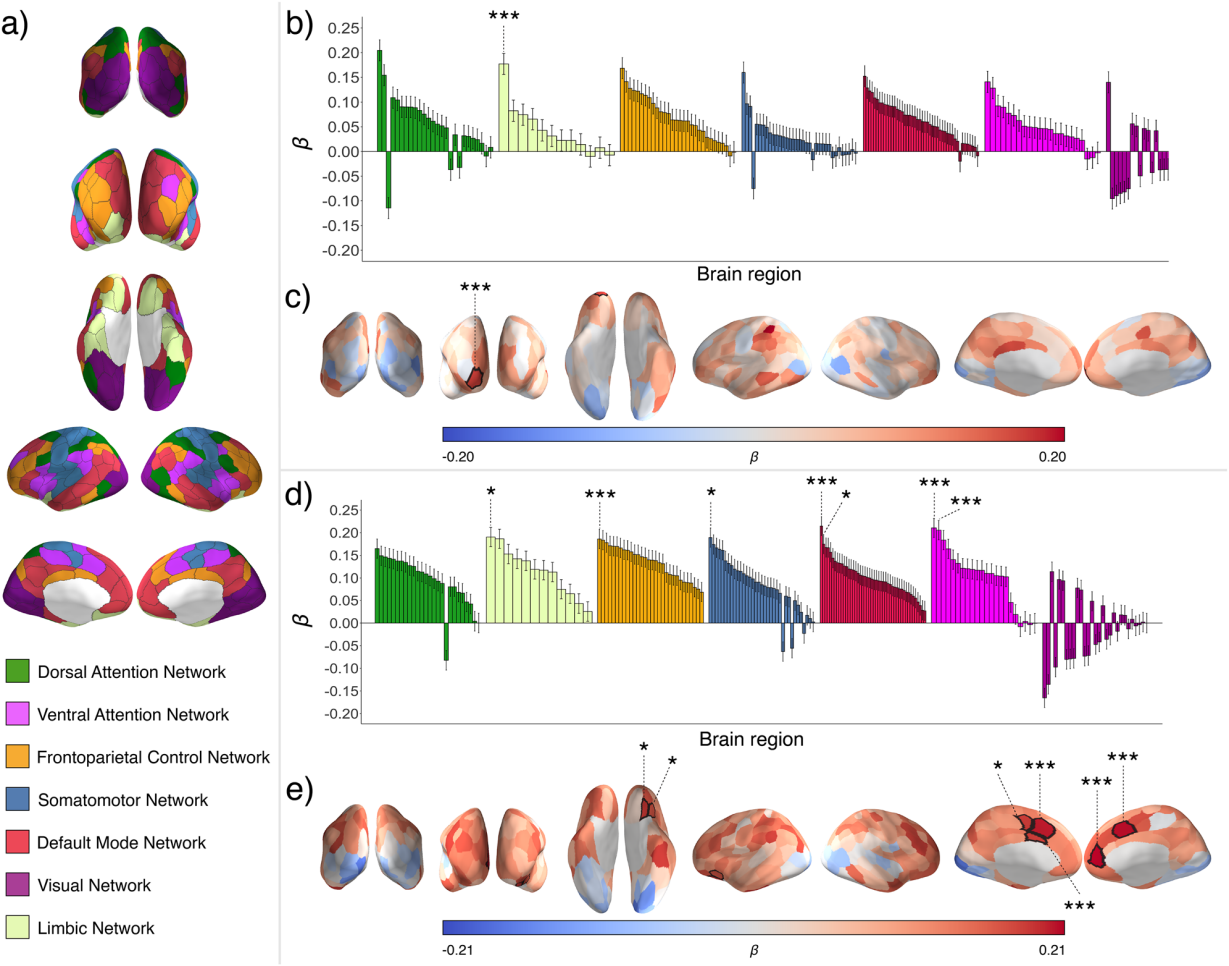
**Whole-brain analyses relating personality similarity and neural synchrony.** (**a**) Time series of neural responses were extracted from each of 200 parcels spanning the cerebral cortex, as well as 14 anatomically-defined subcortical regions (not shown); each cortical parcel was associated with one of seven functional brain networks. These parcels and networks (annotated by different colors) are visualized on a cortical surface model and shown in posterior (top row), anterior (second row), ventral (third row), lateral (fourth row), and medial (fifth row) views. (**b**) Within each brain parcel, the relationship between personality similarity and NM-Synchrony was assessed using OLS regression. One-sample t-tests confirmed that the distribution of effects (other than the Visual Network) significantly differed from zero. (**c**) Regression coefficients for personality similarity are shown overlaid on the inflated cortical surface. Personality similarity was associated with NM-Synchrony in the right OFC. (**d**) The same statistical procedure was repeated to assess the relationships between personality similarity and NP-Synchrony. One-sample t-tests confirmed that the distribution of effects significantly differed from zero in all but the Visual Network. (**e**) Corresponding regression coefficients are shown overlaid on the cortical surface. Personality similarity was associated with NP-Synchrony in the left cingulate cortex, a medial aspect of the left superior frontal cortex, a medial aspect of the left somatomotor cortex, a region of the right medial prefrontal cortex, a medial aspect of the right superior frontal cortex, a region of the left ventrolateral prefrontal cortex, and a region of the left orbitofrontal cortex. Brain parcels where personality similarity was significantly predictive of neural synchrony are marked by asterisks (*p ≤ 0.05, ***p < 0.001, permutation-tested and FDR-corrected).

For each subject, all four fMRI scans were concatenated into a single time series (with the exception of the two subjects who only had three scans that were concatenated). Neural synchrony for each dyad was calculated in the 200 cortical parcels and 14 subcortical brain regions. Neural synchrony was calculated using two metrics: Neural Magnitude Synchrony (NM-Synchrony^4^) and Neural Pattern Synchrony (NP-Synchrony^37,38^). NM-Synchrony captures similarities in spatially-averaged neural response magnitudes over time. To calculate NM-Synchrony, we first extracted the time series of spatially-averaged neural response magnitudes across the entire study for each subject in each of the regions of interest (Fig. 2). We then correlated each unique pair of subjects’ time series within each network. We complemented the NM-Synchrony measure with NP-Synchrony, which examines the similarity in individuals’ spatially distributed neural response patterns over time. The NP-Synchrony measure was added given that widespread evidence has demonstrated the importance of examining not only response magnitudes but also spatially distributed response topographies when characterizing psychological states^39,40^. Average NM-Synchrony and NP-Synchrony in each cortical parcel is visualized in Fig. S2 in the Supplementary Information.

To estimate the reliability of the neural synchrony measures, we split the fMRI scans in half and calculated neural synchrony for each dyad separately based on the first and third period, and based on the second and fourth periods. We subsequently correlated the two similarity measures across all dyads to obtain a measure of split-half reliability. Given that the stimuli varied across the scans and neural synchrony is known to be a function of the content individuals watch^2,41^, the reliability estimates expected for neural synchrony are lower than those expected for traditional psychometric questionnaires (which are typically considered appropriate at *r* = 0.7). Aligned with this expectation, we observed correlations of *r* = 0.26 for NM-Synchrony and *r* = 0.40 for NP-Synchrony.

*Personality similarity*

For each dyad, we estimated personality similarity as the Euclidean distance across the five personality traits:

$${d}_{x,y}=\sqrt{{\sum }_{i=1}^{n}({x}_{i}- {y}_{i}{)}^{2} ,}$$

with *x* = subject 1, *y* = subject 2, and *n* = five personality traits. To ensure the robustness of our findings we tested a number of additional distance measures (using the *proxy* package in *R*). The measures tested include: Manhattan distance ($\sqrt{{\sum }_{i}^{n}|{x}_{i}- {y}_{i}| } )$, Canberra distance $(\sqrt{{\sum }_{i}^{n}\frac{\left|{x}_{i}-{y}_{i}\right|}{\left|{x}_{i}+{y}_{i}\right|} } )$, and Supremum distance (${argmax}_{i}(\left|{x}_{i}-{y}_{i}\right|)$. To facilitate the interpretability of the estimates, we subtracted the metrics from zero such that higher scores on the personality similarity variables indicate higher fit.

We tested the reliability of the personality similarity measure by splitting the personality survey in half (split-half reliability). Within each half, we estimated personality similarity for each dyad. Finally, we calculated the correlation between the two independent similarity estimates across all dyads. The reliability for the derived similarity measures was *r* = 0.27. While this reliability estimate is low, it is not unexpected given that our personality measure was short and limited in its original reliability. Notably, the relatively low reliability of the personality similarity measure introduces an upper bound for the observable relationship between personality similarity and neural synchrony. While some authors recommend reporting relationships corrected for attenuation (i.e., as if their measure was perfectly reliable and free of random noise^42^), we chose to report the true, raw relationships which constitute a conservative estimate of the true effect.

*Socio-demographic similarity*

Gender similarity was coded as 1 = same gender, 0 = opposite gender. Age similarity was calculated as the absolute difference between the age of the two subjects in each dyad. Handedness was coded as 1 = same handedness, 0 = different handedness. Nationality similarity was coded as 1 = same nationality, 0 = different nationality. Ethnicity similarity was calculated as a binary measure indicating overlap in ethnicity (1 = at least one shared ethnicity, 0 = no overlap).

### Results

**Figure 3:**
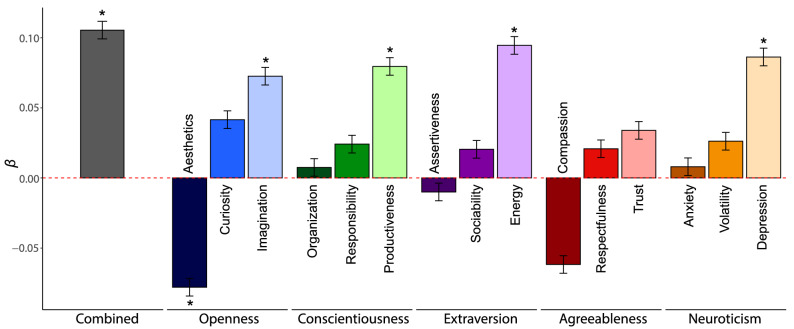
**The relationship between personality similarity and neural synchrony.** Standardized regression coefficients for the overall personality similarity measure as well as for the 15 individual personality facets. The color coding highlights the five main traits, with different shades of the same color distinguishing between the facets (e.g., dark to light blue for the three Openness facets). All coefficients are sorted by effect size within each Big Five trait. Asterisks indicate permutation-tested significance levels with *p < 0.05.

We conducted an exploratory whole-brain analysis to test for relationships between personality similarity and neural synchrony in each of the 200 cortical parcels and 14 subcortical regions of interest. Specifically, we tested whether personality similarity was associated with NM-Synchrony and NP-Synchrony using linear regression analyses with permutation testing. The reported effects are corrected for false discovery rates (FDR) across all regions (Fig. 3).

NM-Synchrony was significantly associated with personality similarity in a region of the limbic network—specifically, the right orbitofrontal cortex (OFC; *ß* = 0.177; *SE* = 0.021; *p* = 1.38 × 10^–16^, *p*_*permutation*_ < 0.001), which remained significant when controlling for inter-subject similarities in gender, age, handedness, nationality, and ethnicity (*ß* = 0.169; *SE* = 0.021; *p* = 2.92 × 10^–15^, *p*_*permutation*_ < 0.001).

NP-Synchrony was significantly associated with personality similarity in a region of the FPCN within the left cingulate cortex (*ß* = 0.186; *SE* = 0.021; *p* = 3.47 × 10^–18^, *p*_*permutation*_ < 0.001), a region of the DMN within the right medial pre-frontal cortex (MPFC; *ß* = 0.215; *SE* = 0.021; *p* = 8.74 × 10^–24^, *p*_*permutation*_ < 0.001), a region of the DMN within the left ventrolateral prefrontal cortex (*ß* = 0.167; *SE* = 0.021; *p* = 7.60 × 10^–11^, *p*_*permutation*_ = 0.029), a region of the limbic network within the left orbitofrontal cortex (*ß* = 0.190; *SE* = 0.021; *p* = 5.91 × 10^–19^, *p*_*permutation*_ = 0.029), a region of the left somatomotor cortex (*ß* = 0.189; *SE* = 0.021; *p* = 9.83 × 10^–19^, *p*_*permutation*_ = 0.029), a region of the ventral attention network within the medial aspect of the left superior frontal cortex (*ß* = 0.205; *SE* = 0.021; *p* = 6.96 × 10^–22^, *p*_*permutation*_ < 0.001), and a region of the ventral attention network within the medial aspect of the right superior frontal cortex (*ß* = 2.12; *SE* = 0.021; *p* = 6.01 × 10^–23^, *p*_*permutation*_ < 0.001).

The majority of these relationships remain statistically significant when controlling for socio-demographic similarity. Specifically, the left cingulate cortex (*ß* = 0.181; *SE* = 0.021; *p* = 2.80 × 10^–17^, *p*_*permutation*_ < 0.001), the right medial prefrontal cortex (*ß* = 0.195; *SE* = 0.021; *p* = 7.53 × 10^–21^, *p*_*permutation*_ = 0.05), the left somatomotor cortex (*ß* = 0.176; *SE* = 0.021; *p* = 0.001, *p*_*permutation*_ = 0.05), and the left ventrolateral prefrontal cortex (*ß* = 0.161; *SE* = 0.021; *p* = 0.001, *p*_*permutation*_ = 0.05) all show significant results when controlling for socio-demographic similarity (after permutation testing; Fig. S2 in the Supplementary Information).

We did not find significant relationships in the 14 subcortical regions of interest (left and right amygdala, thalamus, hippocampus, nucleus accumbens, putamen, caudate, and pallidum).

Consistent with the notion that personality similarity drives mental alignment, the direction of the relationship between personality similarity and neural synchrony was consistently positive across regions (Fig. 2). One-sample t-tests comparing the distribution of coefficients in each network to a Null-distribution confirmed that all distributions for both NM-Synchrony and NP-Synchrony were significantly different from zero (Table S2 in the Supplementary Information). The only exceptions were the coefficients in the visual network. The robust patterns shown across networks and parcels suggest a reliable relationship between personality similarity and neural synchrony.

## Study 2

Study 2 was aimed at: (1) replicating the main effect of personality similarity and neural synchrony using whole-brain EEG analyses, (2) investigating the role of specific personality sub-facets, and (3) examining the underlying perceptual mechanisms driving the effect by complementing the neural signals with eye tracking data.

The methodology used in Study 2 differs from that of Study 1 in several ways. First, Study 2 used discrete images as stimuli, rather than videos. This choice makes our findings more directly comparable to prior eye tracking work and ensures that the eye tracking component of the study is better controlled—with all viewers initiating their gaze at the center. Second, given that the main purpose of Study 2 was to explore the relationship between personality similarity and neural synchrony, we used a much longer and more detailed personality measure that allowed us to investigate the effects of personality facets. Third, to contrast the effects of personality similarity with other personal characteristics that are often discussed in the popular media and that have recently been related to neural synchrony in empirical research^43,44^, Study 2 added political ideology as an additional similarity comparison.

### Methods

All methods were carried out in accordance with the guidelines and regulations put forward by Columbia University. The study protocol in its entirety was approved by Columbia University’s Institutional Review Board.

#### Participants and procedure

Three hundred and three subjects participated in the EEG study and watched a set of 104 images that represent a variety of topics across 26 categories (e.g., people, nature, art). Neural data were acquired using a 32-electrode EEG headset. Gaze data were collected using a stationary eye tracker. Subjects reported their gender, age, ethnicity, and political ideology (Fig. 1) and completed a 60-item personality test^45^ which measures the five broad personality dimensions as well as 15 sub-facets associated with these five traits (e.g., sub-dividing the extraversion trait to the sub-traits sociability, assertiveness and energy).

All subjects had normal or corrected-to-normal vision. We excluded subjects whose EEG signals were noisy in more than 12 of the 32 recording channels or over 10% of the continuous data. The final sample consisted of 225 subjects (60% female; 21.61 ± 5.77 years old). The subjects identified as Asian (56%), Caucasian/White (36%), Black/African (8%), Other (4%) and Native Hawaiian/Pacific Islander or American Indian/Alaska Native (2%, respectively; subjects could identify with multiple ethnicities). All subjects provided written informed consent.

Due to eye tracker malfunction that occurred during data collection, gaze data were available for only 144 of the 225 subjects. All other analyses are based on the full sample of 225 subjects.

#### Materials

Stimuli consisted of 104 images which were downloaded from Shutterstock.com to reflect a broad range of content and styles across 26 categories, four images per category (Table S3 in the Supplementary Information). All images were normed for the eye tracking component of the study. Subjects were told that they would view a “sequence of images, each preceded by a fixation cross that will appear in the middle of the screen”. They were instructed to fixate on the cross and press the *spacebar* to initiate each trial. In each trial an image appeared on the full screen for three seconds, resulting in a total recording time of about 5 min.

#### Measures

##### Personality

We measured subjects’ personality using the 60-item BFI-2 questionnaire^45^. In addition to measuring the five main personality traits, the BFI-2 captures three sub-facets per trait: Intellectual curiosity, aesthetic sensitivity, creative imagination (Openness), organization, productiveness, responsibility (Conscientiousness), sociability, assertiveness, energy (Extraversion), compassion, respectfulness, trust (Agreeableness), and anxiety, depression, emotional volatility (Neuroticism). Given this more granular personality measure, we calculated personality similarity based on 15 facets.

With internal consistencies ranging from α = 0.55 for “responsibility” to α = 0.83 for “organization”, the reliability of the measure was found to be “acceptable” to “excellent” (average 0.72 ± 0.09; see Table S4 in the Supplementary Information for all 15 Cronbach’s alpha values).

##### Neural data

We collected neural data using a 32-channel EEG headset (Brain Products GmbH, Gilching, Germany) at a sampling rate of 500-Hz, and pre-processed the data to correct for instrumental, physiological, and movement-related noise (see Supplementary Information for more details).

##### Eye tracking data

We acquired eye gaze data using a Tobii TX300 eye tracking device (Tobii AB, Danderyd, Sweden; see Supplementary Information for more details). Subjects’ gaze was calibrated using the system’s internal calibration tool (9-point fixation across various locations on the screen, presented in random order, twice, for calibration and error estimate).

##### Similarity measures

*Neural synchrony*

We computed neural synchrony using an adapted version of available code for inter-subject similarity in EEG^46^. The correlation measure is analogous to the fMRI inter-subject synchrony method, while leveraging the high temporal resolution of electrophysiological signals^46–48^. Conceptually, the inter-subject similarity measure projects the raw data onto a component space and seeks to maximize the correlation between two subjects’ components. This analysis is similar to traditional Principal Component Analysis (PCA), except for the fact that it seeks to maximize correlation between two subjects rather than variance. The projection that is most similar within a dyad reflects a transformation of the EEG data that mostly aligns the brain activities. Mathematically, this is implemented by calculating the cross-covariance between- and within-the subjects’ raw EEG channels, and then extracting the eigenvectors of the between-within subject matrix multiplication. The ranked eigenvalues reflect the strength of the correlation between the components, but do not indicate which neural sites drive the correlation between the two brains. We ran our analyses both by looking at each of the top three components individually, as well as their average, in line with prior work^46^. Given that the analyses produced similar results for the three components and their average, we hereafter report the average.

We calculated inter-subject correlation for each image within a dyad. Subsequently, the correlation was averaged across all images for that dyad. In line with the fMRI analyses, we also estimated the reliability of the neural synchrony measure by splitting the stimulus set into odd and even pictures, estimating neural synchrony separately for both sets and calculating the correlation between the two estimates. The reliability score (*r* = 0.71) supports the proposition that neural synchrony is a combination of stable dispositional similarities and variable stimulus-dependent responses.

*Eye gaze similarity*

We used earth-mover distance (EMD) to calculate the similarity in people’s gaze^49^. EMD estimates the distance between two probability distributions over a specific region. Conceptually, EMD is often illustrated as the minimum number of steps one needs to take in order to move a pile of sand (distribution) from one location to another, where it may occupy different space but maintain its number of grains. Translated to the context of eye tracking, one can calculate a probability distribution for each person’s fixations within an image based on the dwell time in various locations within that image. Accordingly, EMD accounts for the number of steps it would take to translate one subject’s fixation data onto another’s. The measure considers both the similarity in fixation location as well as the similarity in fixation duration. For each dyad in our dataset, we calculated the average EMD across all 104 images.

*Personality similarity*

We used the same distance measures as for the fMRI study (Euclidean as the main measure, and Manhattan, Canberra and Supremum as robustness checks). The measures were inverted to have higher scores reflect higher levels of fit.

As for the fMRI analyses, we calculated split-half reliabilities for our personality similarity. Given that the personality measure in Study 2 was based on a longer questionnaire and therefore more reliable, the personality similarity reliability, *r* = 0.66, was also substantially higher than that observed in Study 1.

*Socio-demographic similarity*

We used the same socio-demographic fit measures as in the fMRI study. In addition, we calculated similarity in political ideology as the absolute difference in the response to the question “How conservative or liberal do you consider yourself?” which was rated using a 7-point scale.

### Results

Table S5 in the Supplementary Information includes all zero-order correlations between similarity measures. We estimated the effect of personality similarity on neural synchrony using OLS regression analyses in combination with permutation testing (randomly shuffled across subjects). Personality similarity significantly predicted neural synchrony (*β* = 0.11, *SE(β)* = 0.006, *p* < 0.001, *p*_*permutation*_ = 0.014, Fig. 3), and the effect remained significant when controlling for similarity in age, gender, ethnicity and political ideology (*β* = 0.11, *SE(β)* = 0.006, *p* < 0.001, *p*_*permutation*_ = 0.014). Additional analyses of similarity across the 15 facets showed that the majority of effects were positive (Fig. 3) with four effects remaining significant after permutation testing: imagination (within the openness trait; M1: *β* = 0.07, *SE(β)* = 0.006, *p* < 0.001, *p*_*permutation*_ = 0.041), productiveness (within the conscientiousness trait*;* M1: *β* = 0.08, *SE(β)* = 0.006, *p* < 0.001, *p*_*permutation*_ = 0.018), energy of extraversion (within the extraversion trait; M1: *β* = 0.09, *SE(β)* = 0.006, *p* < 0.001, *p*_*permutation*_ = 0.013), and depression of neuroticism (within the neuroticism trait; M1:*β* = 0.09, *SE(β)* = 0.006, *p* < 0.001, *p*_*permutation*_ = 0.013). The only facet that significantly negatively predicted neural synchrony was the aesthetics facet of the openness trait (M1: *β* =  − 0.08, *SE(β)* = 0.006, *p* < 0.001, *p*_*permutation*_ = 0.032; see General Discussion for a suggested explanation).

#### The mediating role of eye gaze similarity

In addition to replicating the main effect of personality similarity on neural synchrony in the fMRI results, the EEG data afforded an investigation of the effect’s underlying perceptual mechanisms. Specifically, we investigated the mediating role of gaze similarity. We ran OLS regression analyses to estimate the direct and indirect effects of personality similarity and gaze similarity on neural synchrony and account for dyadic dependencies by providing permutation-tested *p*-values for the individual paths.

Gaze similarity did not statistically mediate the relationship between personality similarity and neural synchrony (indirect effect: *β* < 0.001, *SE(β)* < 0.001, *p* = 0.393, *p*_*permutation*_ = 0.425). Neither the effect of personality similarity on gaze similarity (*β* = 0.010, *SE(β)* = 0.010, *p* = 0.315, *p*_*permutation*_ = 0.426) nor the effect of gaze similarity on neural synchrony (*β* = 0.017, *SE(β)* = 0.010, *p* = 0.086, *p*_*permutation*_ = 0.372) were significant. The fact that the relationship between personality similarity and neural synchrony was not mediated by gaze similarity provides suggestive evidence that it might be driven by differences in how people encode and interpret external stimuli, rather than by differences in how they focus their overt attention. For example, extraverts’ and introverts’ attention might both be drawn to the same aspects of a stimulus (e.g., a group of people), but their response to that stimulus is likely to differ (e.g., approach versus avoidance).

## Studies 1 and 2: robustness checks

**Figure 4:**
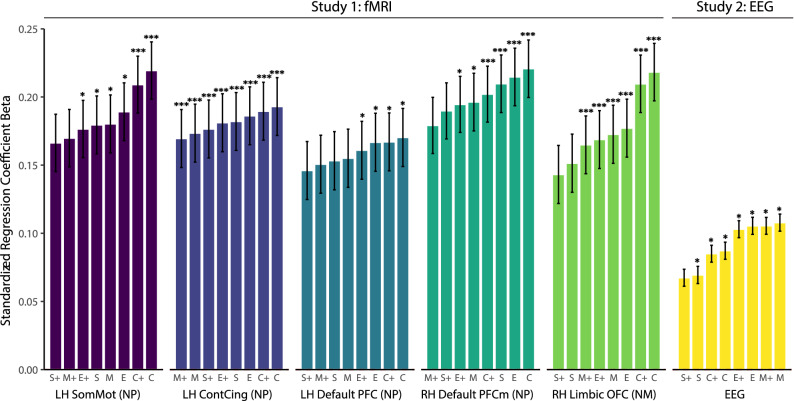
**Estimates of the relationship between personality similarity and neural synchrony using four different similarity metrics. **The relationship between personality similarity and neural synchrony was consistent across coefficients and multiple model specifications (4 personality similarity measures × 2 models, one with- and one without controls). All coefficients are standardized effects sorted by effect size within each brain region for which significant effects were found in the fully controlled model (fMRI) as well as for the whole brain analyses (EEG). *LH* left hemisphere, *RH* right hemisphere, *NM* neural magnitude synchrony, *NP* neural pattern synchrony, *SomMot* somatomotor cortex, *ContCing* cingulate cortex parcel within frontoparietal control network, *Default PFC* prefrontal cortex parcel within default mode network, *Default PFCm* medial prefrontal cortex parcel within default mode network, *Limbic OFC* orbitofrontal cortex parcel within limbic network. The coefficients are labelled with the respective reverse-coded distance measure, with *E* Euclidean, *M* Manhattan, *C* Canberra, and *S* Supremum. The + sign denotes models that include the socio-demographic similarity measures as control variables. Asterisks indicate permutation-tested and FDR corrected significance levels with *p < 0.05, ***p < 0.001.

We conducted a series of robustness checks to support the internal validity of our findings. First, we tested the main effect of personality similarity on neural synchrony using three additional personality similarity metrics (Manhattan, Canberra, and Supremum distances; all reverse coded) to ensure that the effects are not driven by the properties of the Euclidean distance measure. The effects were stable when estimating the different model specifications, highlighting the robustness of the effect (Fig. 4). All distributions were significantly different from zero (*p* < 0.001).

**Figure 5:**
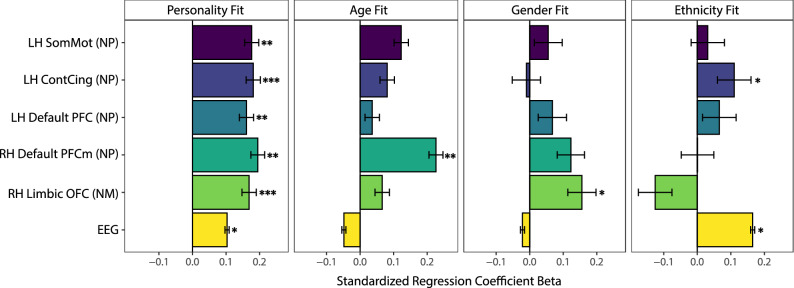
**Effect sizes comparison.** Comparing effect sizes between personality similarity and all socio-demographic measures available in both fMRI and EEG (using the main Euclidean distance similarity metric). *LH* left hemisphere, *RH* right hemisphere, *NM* neural magnitude synchrony, *NP* neural pattern synchrony, *SomMot* somatomotor cortex, *ContCing* cingulate cortex parcel within frontoparietal control network, *Default PFC* prefrontal cortex parcel within the default mode network, *Default PFCm* medial prefrontal cortex parcel within the default mode network, *Limbic OFC* orbitofrontal cortex parcel within the limbic network. Asterisks indicate permutation-tested significance levels with *p < 0.05, **p < 0.01, ***p < 0.001.

Second, we benchmarked our findings with respect to effect size by comparing the effect of personality similarity to the effect of similarity on other socio-demographic factors: gender, age and ethnicity. Personality similarity was found to be a statistically stronger predictor of neural synchrony than similarity in gender (*t*(10) = 3.25, *p* = 0.009) and ethnicity (*t*(10) = 2.86, *p* = 0.017)—and marginally stronger than age (*t*(10) = 2.11, *p* = 0.061)—when compared across all six focal effects (see Fig. 5 for a visual depiction of the effect sizes that were compared). In the EEG data, personality similarity also outperformed similarity in political ideology (contrast analysis within the EEG model: *p* < 0.001), which was found to be non-significant. Personality was the only predictor that showed consistent positive results across all fMRI and EEG measures.

**Figure 6:**
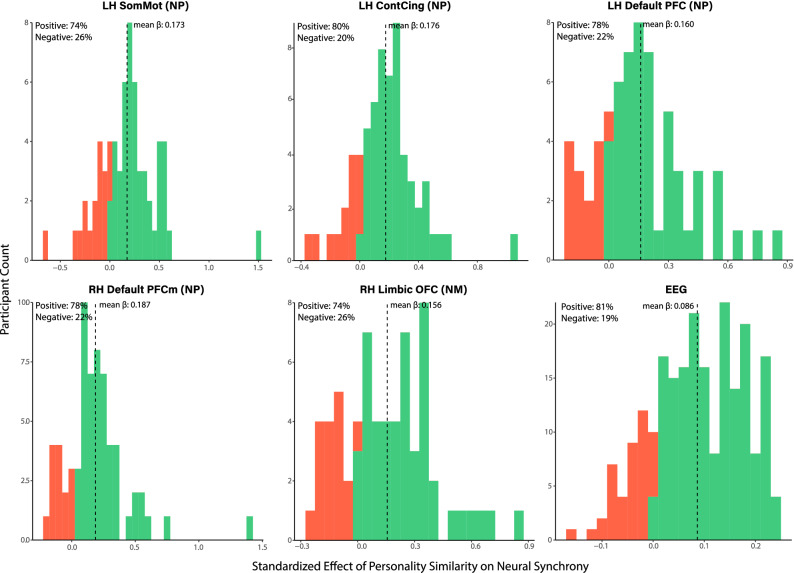
**Distribution of effects across the sample.** Distribution of standardized regression coefficients capturing the relationship between personality similarity and neural synchrony for each subject in the fMRI and EEG samples. Positive coefficients (green) indicate a positive link between personality and neural synchrony for that individual while negative coefficients (red) indicate a negative link. *LH* left hemisphere, *RH* right hemisphere, *NM* neural magnitude synchrony, *NP* neural pattern synchrony, *SomMot* somatomotor cortex, *ContCing* cingulate cortex parcel within the frontoparietal control network, *Default PFC* prefrontal cortex parcel within the default mode network, *Default PFCm* medial prefrontal cortex parcel within the default mode network, *Limbic OFC* orbitofrontal cortex parcel within the limbic network.

Finally, we estimated the relationship between personality similarity and neural synchrony separately for each subject (controlling for all additional variables such as age, gender, etc.) to test whether the relationship could be observed for the majority of subjects. That is, we iteratively subset our data to a single subject and ran the regression analyses on the observations of that particular individual (n_obs_/person = 65 for the fMRI and n_obs_/person = 224 for the EEG. Showing that the effects are not merely driven by a small number of individuals, we found a positive effect of personality similarity on neural synchrony for the vast majority of subjects (ranging from 74 to 81% positive; Fig. 6).

## General discussion

A shared perspective on the social and physical world is critical for fostering successful communication and cooperation among different members of society. Much of the contemporary public discourse on shared perspectives is focused on the extent to which homogeneity in surface-level characteristics such as age, gender, ethnicity or political ideology leads groups of individuals to adopt similar perspectives and outlooks on the world. Offering a more nuanced perspective, our findings from fMRI and EEG analyses provide robust evidence that the extent to which people are aligned in their neural activity when viewing naturalistic stimuli depends on how similar they are in their psychological dispositions. In fact, the observed effects of personality similarity on neural synchrony were stronger than those for socio-demographic similarity, suggesting that when it comes to aligning our experiences of everyday naturalistic stimuli (e.g., viewing an image of a group of friends or a video clip of a debate about college football), similarity in people’s deep level personality traits is more important than similarity in surface level socio-demographic characteristics^50^.

In our fMRI study, personality similarity was associated with similar neural responses in brain regions implicated in high-level processes, such as how people interpret and emotionally respond to their environment. Specifically, personality similarity was associated with neural synchrony in brain regions associated with subjective construal, (e.g., the MPFC region within the DMN^6^). This may reflect similarity in interpretations of the content viewed by subjects and could stem from similar biases and priors in construing incoming information among people with resembling personalities. Additionally, personality similarity was associated with aligned neural responses in the left cingulate cortex within the FPCN, which is implicated in cognitive control and decision-making processes^51^, and which is thought to couple with the DMN to generate internal trains of thought^52^. These processes are essential for understanding complex narratives (e.g., a movie plot), a process that involves remembering past events, interpreting current events, predicting future events, and integrating those processes across time^53^. The current results suggest such processes may ebb and flow over time similarly in people with similar personalities. Additionally, past fMRI work has shown that when watching movies, individuals with autism spectrum disorder exhibit exceptionally low neural synchrony in the FPCN relative to neurotypical individuals^54^, and more generally, that similarity in subjective understanding of stimuli is associated with greater neural synchrony in the FPCN^55^. Together, these suggest that inter-individual variation in FPCN activity reflects differences in how people process naturalistic stimuli, and in particular, how processing of such stimuli unfolds over time. Thus, localization of the current results in the left cingulate cortex within the FPCN suggest that individuals with similar personalities are likely to share exceptionally aligned experiences when viewing naturalistic stimuli. Personality similarity was also related to neural synchrony in regions associated with subjective value, e.g., the OFC^56^, which may reflect alignment of tastes and preferences (e.g., what an individual considers funny).

The observed relationships between personality similarity and neural synchrony in our whole-brain analyses are particularly robust, given that they remained significant using a conservative permutation testing process that was implemented in combination with FDR correction across 214 brain regions. Taken together, the current results suggest that personality similarity is associated with similarity in the spontaneous deployment of processes related to subjective value and interpretation during naturalistic stimulation.

The EEG analyses support the association between personality similarity and neural synchrony by showing that the effect is robust when shifting from high spatial to high temporal resolution. In addition, the analyses provide deeper insights into the nuances of personality that drive neural synchrony. While similarity in nearly all personality facets was positively associated with neural synchrony, the effects were found to be significant only for imagination (Openness), productiveness (Conscientiousness), energy (Extraversion), and depression (Neuroticism). Although the interpretation of any individual effect remains speculative in the absence of replication, the importance of these facets could be explained in a number of ways. Depression, for example, is the Neuroticism facet that likely produces the most stable responses when it comes to interpreting external stimuli. While anxiety might be context-dependent, and volatility does not necessarily mean that two individuals will have the same reaction at the same time, depression is likely to create a generally negative interpretation of the external world. Similarity in imagination and energy, on the other hand, might drive neural synchrony by determining the extent to which subjects create concrete interpretations of the stimuli in their mind and, generally, exhibit strong responses to the input. The aesthetics facet (Openness) was the only instance for which a significantly negative effect of personality similarity on neural synchrony was observed. This finding might be explained by the unique nature of the aesthetics facet. While high similarity scores indicate a shared interest in aesthetics, this does not mean that the aesthetic preferences of two individuals are the same. Rather, it is conceivable that high scores of aesthetics divergence indicate that the person has a unique taste. This interpretation aligns with the fMRI results showing the notable activity in regions that are implicated in subjective preferences are dominant in driving the synchrony.

Finally, the additional analyses of eye tracking data suggest that neural synchrony among people with similar personalities is not driven by selective attention but rather by the intrinsic response to the content. Even though the EEG methodology did not explicitly test for neural synchrony in the visual attention areas, the fact that we did not observe a significant relationship between personality similarity and eye gaze similarity suggests that the effect is unlikely to be driven by subjects overtly attending to the same aspects of the stimuli. Together with the results of the fMRI study, which showed significant effects in areas associated with subjective values and interpretations of external stimuli (i.e., DMN, FPCN and OFC) but not in areas associated with visual attention, the eye tracking findings support the proposition that the relationship between neural synchrony and personality similarity may stem from similarities in higher-level processes, such as how people interpret their environment and emotionally respond to it.

There are a number of important limitations associated with this research. First, the paradigms used in the two studies differed in several important ways. This included the type of stimuli (video versus images), the length of the personality assessments (with only Study 2 allowing for the analysis of personality facets), the method for capturing neural responses (EEG versus fMRI), and—as a consequence—the analyses used to examine the data. The difference in methodologies means that the findings from Study 1 and Study 2 are not directly comparable. However, while this is partly a limitation of the current research, the multimodal, multimethod approach also strengthens the generalizability of our findings by conceptually (rather than directly) replicating them. While each of the methods has its own advantages and limitations^31,32^, together they provide robust evidence for the relationship between personality similarity and neural synchrony and its underlying mechanisms.

Second, the fMRI results were limited by the short personality questionnaire, which resulted in low reliabilities of the traits estimates and the personality similarity measures. This measurement error makes it more difficult to detect true relationships between personality similarity and neural synchrony. Hence, it is possible that future research which uses more reliable personality measures would uncover additional significant effects in other brain regions.

Third, the work did not directly test the hypothesis that personality similarity is linked to neural synchrony through a shared interpretation of stimuli, but rather infers this proposition from the combination of the eye tracking and fMRI results. Future work should test this theory directly (e.g., by surveying individuals about their interpretations).

Fourth, our analyses focused on a wide range of stimuli that people might be exposed to in daily life. While this approach provides us with an estimate of the importance of personality similarity across a variety of daily experiences, given that stimuli that elicit particularly strong affective responding evoke particularly reliable neural responses^3^, prioritizing such stimuli could enhance sensitivity in detecting links between personality and neural synchrony. In addition, it is possible that the neural circuits engaged in the alignment of perspectives change as a function of the specific stimuli chosen. Thus, future work that adopts design and data analytic approaches that support testing for links between personality similarity and neural synchrony independently across stimuli, allowing the particular brain regions where personality similarity is linked to synchrony to vary across stimuli, could afford sensitivity to broader links between personality similarity and neural synchrony.

Fifth, we only focused our analyses on university students who are likely to share the same context and experience of external stimuli more than two randomly drawn strangers from the general population. While this restriction is likely to lead to an underestimation of the effects, future research should test the generalizability of the findings in other populations.

Finally, we did not investigate the behavioral consequences of the neural synchrony facilitated by personality similarity. Future research could test whether neural synchrony leads to real-life consequences by impacting people’s alignment in behavior. In other words, does a shared representation of a scene evoke similar behavior (e.g., do people who have a similar response to observing a homeless person show similar helping behavior?).

Our findings support the growing body of literature suggesting that psychological traits are not only reflected in brain structure, but also in how individuals perceive and interpret the world^11^. The findings suggest that the extent to which we are similar in our personality is a stronger predictor of whether we experience the world in a similar way than some of the commonly investigated socio-demographic variables, including gender, ethnicity, and political ideology. While we have focused our investigation on how personality similarity is linked to neural synchrony as a measure of passive synchronization, future research should explore how personality similarity is associated with active alignment in perspectives in the moment when individuals have the opportunity to experience their physical and social environment together and communicate with one another^13^.

It has not escaped our notice that as we continue to understand the neural foundations of personality, we might be able to relate many of the consequential outcomes that have previously been related to personality^18^ to people’s neural responses to their environments. In addition, insights from personality psychology could be used to understand heterogeneity in the neural activity observed in response to natural stimuli, which is often discarded as noise and measurement error^57^. By observing heterogeneity along a number of well-established psychological dimensions, researchers can tap into the wealth of existing knowledge on psychology to interpret their findings and generate new hypotheses.

## Supplementary Information

Supplementary Information.

## Data Availability

Pre-processed EEG and fMRI data, survey data and analyses scripts are available on OSF and can be accessed via the following link: https://osf.io/ycde9/?view_only=81999d08bab64a879a5214db36964889. Raw data are available from the corresponding author on reasonable request.
