# Supplementary material for: Personality similarity predicts synchronous neural responses in fMRI and EEG data
Source: Sci Rep. 2022 Aug 22;12:14325. doi: 10.1038/s41598-022-18237-1 (PMC9395427; doi:10.1038/s41598-022-18237-1)
Supplement: Supplementary file 1 — Supplementary Information. [file 41598_2022_18237_MOESM1_ESM.docx]

**Supplementary Information**

**fMRI Data Acquisition and Preprocessing**

Neuroimaging data were acquired using a 3-Tesla Siemens Prisma scanner with a 32-channel head coil. An echo-planar sequence (37 ms echo time, TE; 800 ms repetition time, TR; 2.0 mm *x* 2.0 mm *x* 2.0 mm resolution; 104 *x* 104 mm matrix size; 208 *x* 208 field of view, FOV), 72 interleaved slices with no gap, multi-band acceleration factor 8, and 2.0 mm slice thickness) were used to acquire the functional images. Stimuli were presented across four functional scans to allow experimenters to check that the subjects remained awake and comfortable throughout the study; scans were presented in the same order to all subjects. In total, the videos were 59 minutes and 37 seconds in length. A high-resolution T1-weighted (T1w) anatomical scan was acquired for each subject (1,900 ms TR; 2.48 ms TE; 256 *x* 256 mm matrix size; 256 *x* 256 mm FOV; 1.0 mm *x* 1.0 mm *x* 1.0 mm resolution; 208 interleaved slices with 0.5 mm gap; 1.0 mm slice thickness) at the end of the scanning session. Adhesive tape was attached to the headcase and stretched across subjects’ foreheads to minimize head motion.

fMRIPrep version 1.4.0 was used for anatomical and functional data preprocessing ^1^. Each subject’s T1w image was corrected for intensity non-uniformity with N4BiasFieldCorrection, distributed with ANTs 2.1.0, and used as T1w-reference throughout the workflow. The T1w-reference was then skull-stripped with a Nipype implementation of the antsBrainExtraction.sh workflow (from ANTs), using OASIS30ANTs as target template. Spatial normalization to the ICBM 152 Nonlinear Asymmetrical template version 2009c (MNI152NLin2009cAsym) was performed through nonlinear registration with antsRegistration (implemented in ANTs 2.1.0), using brain-extracted versions of both T1w volume and template. Brain tissue segmentation of cerebrospinal fluid, white-matter and gray-matter was performed on the brain-extracted T1w using FSL FAST.

For each of the four scans, per subject, the following preprocessing was performed. First, a reference volume and its skull-stripped version were generated using a custom methodology of fMRIPrep. A BOLD reference was then co-registered to the T1w reference using FSL FLIRT with boundary-based registration cost-function. Co-registration was configured with nine degrees of freedom to account for distortions remaining in the reference. Head-motion parameters with respect to the reference (transformation matrices, and six corresponding rotation and translation parameters) were estimated before any spatio-temporal filtering using FSL MCFLIRT. Automatic removal of motion artifacts using independent component analysis was performed on the preprocessed BOLD in MNI space time series after removal of non-steady state volumes and spatial smoothing with an isotropic, Gaussian kernel of 6 mm full-width at half-maximum. The BOLD time series were then resampled to MNI152NLin2009cAsym standard space, generating a preprocessed BOLD signal in MNI152NLin2009cAsym space.

The confounding variables generated by fMRIPrep that were used as nuisance variables in the current study included global signals extracted from within the cerebrospinal fluid, white matter, and whole-brain masks, framewise displacement, three translational motion parameters, and three rotational motion parameters.

**fMRI Similarity Indices**

In the exploratory analysis, neural synchrony, for each dyad, was calculated in 200 cortical and 14 subcortical brain regions. In each of the 214 regions, the mean fMRI response across all voxels was extracted at each time point (i.e., at each TR), resulting in a response magnitude time series. For each of the 2,145 unique dyads and each of the 214 brain regions, we calculated the Pearson correlation between subjects’ mean response time series, and then applied Fisher-*z* transformation for correlation coefficients (i.e., inverse hyperbolic tangent function). This process yielded a measure reflecting the inter-subject similarity in response magnitude time series across the whole study (NM-Synchrony). Outliers were defined as values outside of the 1.5 interquartile ranges (IQRs). Disproportionately high similarity values were assigned the value of the third (upper) quartile plus 1.5 times the IQR. Disproportionately low similarity values were assigned a value equal to the first (lower) quartile minus 1.5 times the IQR. To calculate NM-Synchrony in a given brain network, NM-Synchrony values in the cortical regions associated with the brain network were averaged to yield a single NM-Synchrony value. This procedure was used to calculate NM-Synchrony in the DAN, FPCN, and DMN. Similarity values associated with each brain network and with each brain region were normalized using Scikit-learn’s *RobustScaler* function ^2^.

Widespread evidence has demonstrated the importance of examining not only response magnitudes, but also spatially distributed response topographies, for characterizing psychological states ^3,4^. Thus, we also calculated inter-subject similarities in multi-voxel response pattern time series (NP-Synchrony). For each subject, in each of the cortical parcels and subcortical sites, multi-voxel response patterns were extracted at each time-point. Pearson correlations between multi-voxel response patterns at each pair of time-points were calculated to construct a time-point *x* time-point matrix that captures the trajectory of multi-voxel response patterns over time within each brain region within each subject. Each element of this matrix reflects the degree to which multi-voxel response patterns are correlated at each pair of time points. For each of the 2,145 dyads, we calculated the Pearson correlation between subjects’ temporal trajectories of multi-voxel response patterns. These values were Fisher-*z* transformed to yield a measure reflecting the inter-subject similarity in multi-voxel response pattern time series across the whole study. Outlier reassignment and data scaling were conducted as described above.

1. **Supplementary fMRI Methods and Results**

**Table S1. Description of fMRI stimuli**

| **Video** | **Content** | **Seconds** |
| --- | --- | --- |
| An Astronaut’s View of Earth | An astronaut discusses viewing Earth from space, and in particular, witnessing the effects of climate change from space. He then urges viewers to mobilize to address this issue | 223 |
| All I Want | A sentimental music video depicting a social outcast with a facial deformity seeking companionship | 305 |
| Scientific demonstration | An astronaut at the International Space Station demonstrates and explains what happens when one wrings out a waterlogged washcloth in space | 119 |
| Food Inc. | An excerpt from a documentary discussing how the fast food industry influences food production and farming practices in the United States | 178 |
| We Can Be Heroes | An excerpt from a mockumentary-style series in which a man discusses why he nominated himself for the title of Australian of the Year | 202 |
| Ban College Football | Journalists and athletes debate whether or not football should be banned as a college sport | 195 |
| Soccer match | Highlights from a soccer match | 91 |
| Ew! | A comedy skit in which grown men play teenage girls disgusted by things around them | 169 |
| Life’s Too Short’ | An example of ‘cringe comedy’ in which a dramatic actor is depicted unsuccessfully trying his hand at improvisational comedy | 106 |
| America’s Funniest Home Videos’ | A series of homemade video clips depicting examples of unintentional physical comedy arising from accidents | 101 |
| Zima Blue | An animated, philosophical short set in a futuristic world | 508 |
| Nathan For You | An episode from a docu-reality comedy in which the host convinces people, who are not always in on the joke, to engage in a variety of strange behaviors | 734 |
| College Party | An excerpt from a film depicting a party scene in which a bashful college student is pressured to drink alcohol | 210 |
| Eighth Grade | Two excerpts from a film depicting a young teenager publicly video blogging about her mental health issues and an awkward scene between two teenagers having a dinner date. | 436 |

**
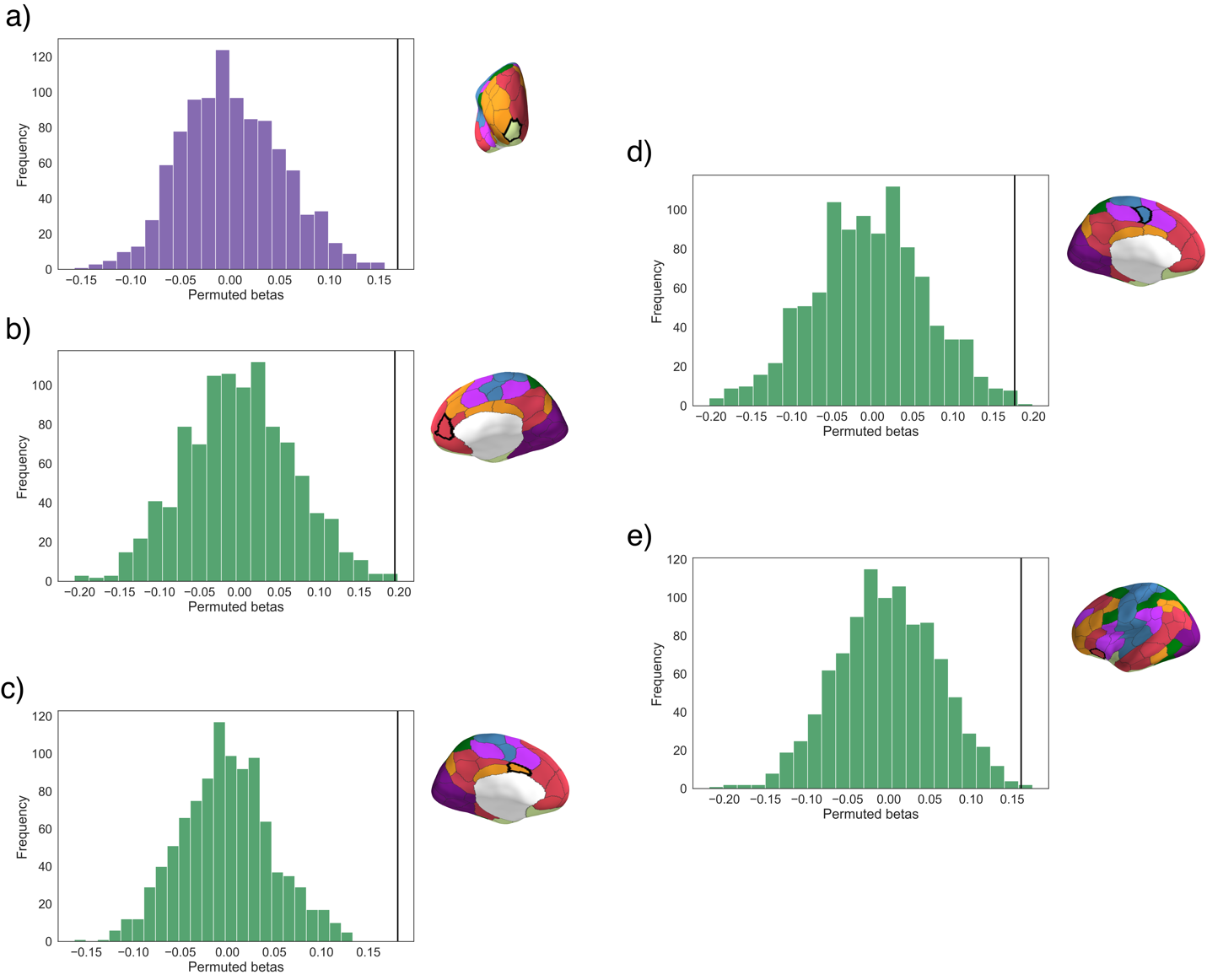
 Figure S1. Permutation testing histograms for statistically significant relationships between personality similarity and neural synchrony when controlling for socio-demographic variables.** For each statistical test, corresponding Null distributions of sham beta coefficients for personality similarity were generated via permutation testing. The resulting Null distributions are shown relative to the true beta coefficient for personality similarity (depicted by the vertical black line) when predicting neural synchrony while controlling for socio-demographic variables in the a) right orbitofrontal cortex within the Limbic network, b) right MPFC within the DMN, c) a portion of the left cingulate cortex within the FPCN, d) a medial portion of left somatomotor cortex, and e) a portion of the ventrolateral prefrontal cortex within the DMN.

**
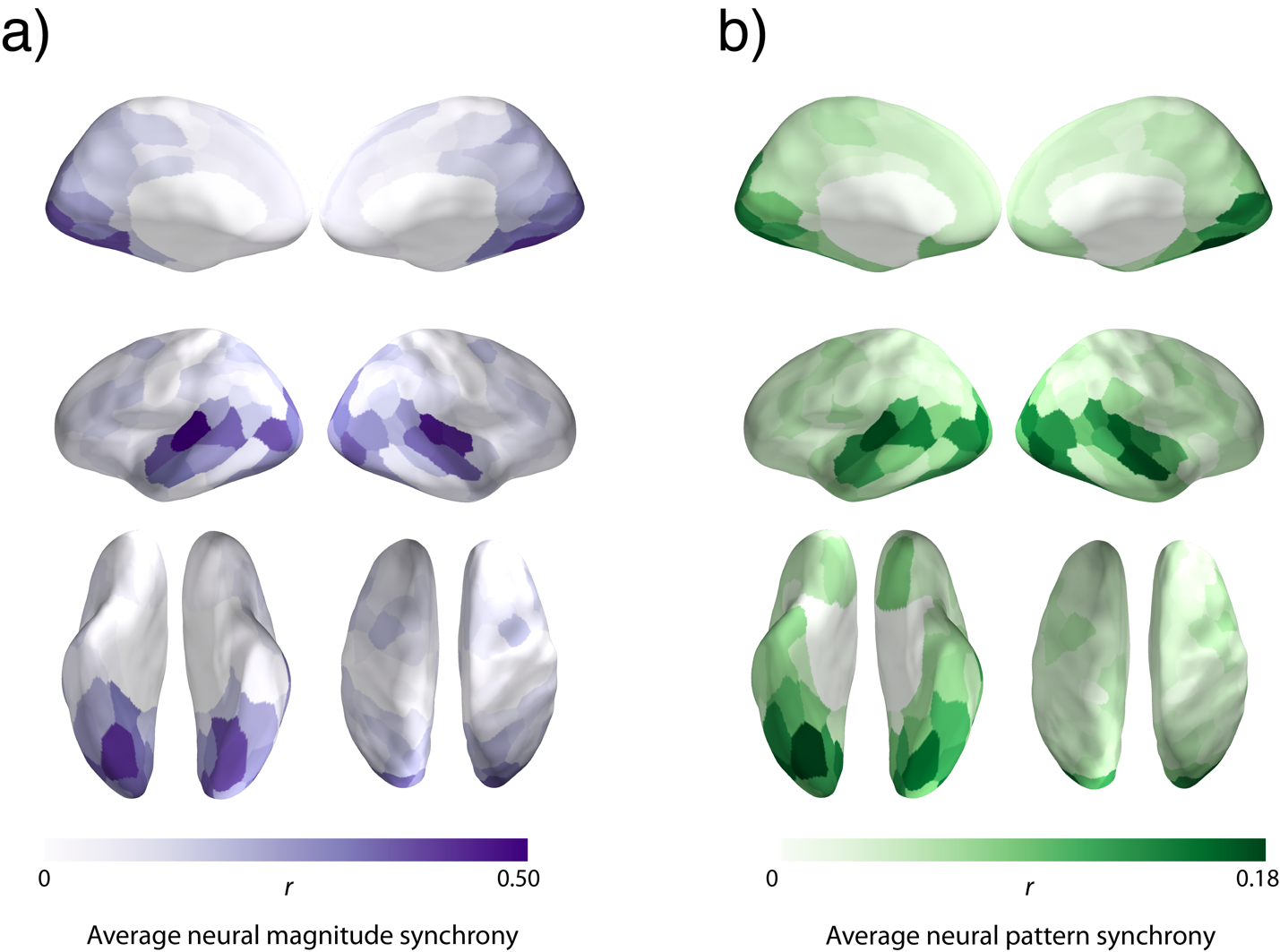
**

**Figure S2. NM-Synchrony and NP-Synchrony averaged across all dyads.** a) NM-synchrony was calculated for each dyad in each of the 200 cortical parcels, and these correlation values were then averaged across all dyads. b) NP-synchrony was calculated for each dyad in each of the 200 parcels, and these correlation values were then averaged across all dyads. These correlation values are visualized on inflated cortical surface models.

**Table S2. Testing if model coefficients significantly differ from zero**

| **Synchrony** | **Brain network** | **Degrees of freedom** | **t-value** | **p-value** |
| --- | --- | --- | --- | --- |
| NM | Limbic | 11 | 2.92 | 1.39$\cdot$10^-2^ |
| NM | Somatomotor | 34 | 3.79 | 5.94$\cdot$10^-4^ |
| NM | Dorsal attention | 25 | 4.21 | 2.89$\cdot$10^-4^ |
| NM | Frontoparietal control | 29 | 8.34 | 3.43$\cdot$10^-9^ |
| NM | Default mode | 45 | 11.02 | 2.27$\cdot$10^-14^ |
| NM | Ventral attention | 21 | 5.98 | 6.24$\cdot$10^-6^ |
| NM | Visual | 28 | -1.26 | 0.219 |
| NP | Limbic | 11 | 7.46 | 1.26$\cdot$10^-5^ |
| NP | Somatomotor | 34 | 7.87 | 3.62$\cdot$10^-9^ |
| NP | Dorsal attention | 25 | 8.50 | 7.72$\cdot$10^-9^ |
| NP | Frontoparietal control | 29 | 20.73 | 6.17$\cdot$10^-19^ |
| NP | Default mode | 45 | 17.64 | 7.62$\cdot$10^-22^ |
| NP | Ventral attention | 21 | 7.21 | 4.17$\cdot$10^-7^ |
| NP | Visual | 28 | -0.97 | 0.339 |

**Supplementary EEG Methods and Results**

**Table S3.** **Image categories of the stimuli used in the EEG Study**

| 1. Abstract | 8. Editorial | 15. Interiors | 22. Science |
| --- | --- | --- | --- |
| 2. Animals | 9. Education | 16. Miscellaneous | 23. Sports |
| 3. Arts | 10. Food | 17. Nature | 24. Technology |
| 4. Backgrounds / Texture | 11. Healthcare & Medical | 18. Objects | 25. Transportation |
| 5. Beauty | 12. Travel | 19. Parks | 26. Vintage |
| 6. Buildings | 13. Illustrations | 20. People |  |
| 7. Business | 14. Industrial | 21. Religion |  |

**Neural Data**

***Data Acquisition***

Neural data were collected using a 32-channel EEG system (Brain Products GmbH, Gilching, Germany) at a sampling rate of 500 Hz. The acquisition system, Brain Vision LiveAmp, had a measurement range of ± 341.6 mV with a gain factor of 12. The device used an internal battery (capacity: 1,000 mAh; approximately 4 hours of recording at 500 mA) that was charged before the experiment to reduce line noise. The device itself applied a common-mode rejection for artifacts greater than 80 dB, and a low-pass filter inside the amplifier of 131 Hz. The data were recorded as 24-bit samples with a resolution of approximately 40.7 nano-Volt per bit. Beyond the 32-channels EEG data we collected three additional 3-axis acceleration data, which were used for artifact rejection based on notable head movements.

Subjects were fitted with an EEG electrode cap with a circumference of either 54 or 58 centimeters, based on head size. The electrode locations were distributed across the entire scalp according to the actiCap 32Ch Standard-2 (green holders) montage (Brain Products GmbH, Gilching, Germany), which correspond to the 10-20 International system (**Figure S3**). The electrodes used were either flat ones touching the surface of the skin – primarily for frontal sites – or pin-electrodes with flexible tip that were made to penetrate the hair. For the pin-electrodes, if neither a 12 nor 14 mm electrode yielded a clear signal upon visual inspection, a washable conductive gel was applied at the electrode tip to improve the signal quality. Subjects used a chin-rest for the duration of the experiment to reduce head movement.


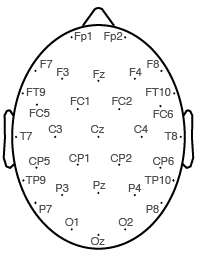


**Figure S3.** Electrode locations (montage) used in the experiment (10-20 international system).

***Preprocessing***

Data were pre-processed using Matlab’s EEGLab extension. None of the pre-processing was done manually to allow for reproducibility. The random number generators implemented in the artifact removals functions of EEGLab were reset to a common seed to remove any randomness. To process the data we first applied a low-pass filter at 0.75 Hz and a high-pass filter at 50 Hz (function: *pop_eegfiltnew*). Filtering was done using Hamming windows Finite Impulse Response (FIR) filter. We subsequently used the *clean_artifacts* function to remove artifacts using the following criteria:

- Maximum tolerated flatline duration of 5 seconds. That is, if a channel had a longer flatline than 5 seconds then the period was considered abnormal.
- Minimum channel correlation of 0.85. That is, if two channels shown a correlation lower than 0.85 in certain periods then the period was considered abnormal.
- Line noise standard deviation of 4. That is, if a channel had 4 standard deviations more of line noise than the relative signal in a certain period, based on the total channel data, the period was considered abnormal.
- An aggressive (strict) burst criterion of 5, indicating that data portions whose variance was larger than 5 standard deviations relative to the median was considered an artifact and were removed.

Portions of the data that had continuous samples were flagged as abnormal and rejected. Channels that had more than 10% of the data flagged as artifacts were marked as unusable. Subjects who had more than 12 channels out of the 32 discarded were excluded from further analyses. If data from a certain electrode were missing or discontinued during the recording, the electrical activity at that site was interpolated by calculating a weighted average of signals from nearby functioning electrodes using the *pop_interp* function. We used spherical interpolation only if at least three nearby electrodes did not include removed data in the specific portion. Finally, we referenced the data to an average reference across all remaining electrodes.

**Eye Tracking Data**

Gaze data were acquired using a Tobii TX300 eye-tracking device (Tobii AB, Danderyd, Sweden). Stimuli were displayed on a 23 inch monitor with a screen resolution of 1920 *x* 1080 pixels and a refresh rate of 60 Hz. Screen luminance was 300 cd/m^2^, and the room lighting was kept constant across all subjects. In addition, the screen distance from the monitor was kept constant by using a chin-rest fixed at a distance of 26 inch. This yields a vertical visual angle of 35 degress from the center for the screen.

The eye-tracker consisted of two sensors located at the bottom of the monitor. Gaze data were sampled at 250 Hz. Fixations were defined by the acquisition software (Tobii Studio 3.4.8.1348) at a window length of 20 ms with an I-VT classifier for 30 degrees/second. Minimal fixation duration was set to 60 ms. Adjacent fixations with an angle of up to 0.5 degrees and 75 ms apart were merged.

To calibrate the eye-tracker, each subject had to fixate on nine locations on the screen ranging from top-left to bottom-right. The software ranked the distance from the fixation location. If the software score was less than “Good” we repeated the calibration after aligning the subject location and the screen rotation. Data were recorded using the software’s proprietary format, and included the *x,y* coordinates of the eyes at each timestamp *t*. We analyzed data only from the right eye across all subjects. Prior to each trial a white fixation cross on a grey background appeared on the screen, which allowed for a future estimation of the calibration error for each subject if the data were showing calibration drifts.

**Table S4.** **Cronbach’s alpha for personality facets**

| **Facet** | **Cronbach’s alpha** |
| --- | --- |
| Intellectual curiosity (O) | 0.68 |
| Aesthetic sensitivity (O) | 0.69 |
| Creative imagination (O) | 0.74 |
| Organization (C) | 0.83 |
| Productiveness (C) | 0.74 |
| Responsibility (C) | 0.55 |
| Sociability (E) | 0.82 |
| Assertiveness (E) | 0.72 |
| Energy (E) | 0.74 |
| Compassion (A) | 0.57 |
| Respectfulness (A) | 0.66 |
| Trust (A) | 0.66 |
| Anxiety (N) | 0.78 |
| Depression (N) | 0.82 |
| Volatility (N) | 0.80 |

**Zero-order correlations**

**Table S4** depicts the Pearson zero-order correlation across the focal variables used in the EEG study analyses. All correlations are based on data from 225 subjects (25,200 dyadic observations). The only exceptions are the correlations for eye gaze similarity, which are based on data from 138 subjects (10,440 dyadic observations).

**Table S5.** **Pearson zero-order correlations and confidence intervals.**

| **Similarity** | **1** | **2** | **3** | **4** | **5** |
| --- | --- | --- | --- | --- | --- |
| 1. Neural | - |  |  |  |  |
| 1. Gaze | 0.017  [-0.002, 0.035] | - |  |  |  |
| 1. Personality | 0.105  [0.093, 0.118] | 0.010  [-0.009, 0.029] | - |  |  |
| 1. Gender | -0.018  [-0.031, -0.006] | 0.042  [0.030, 0.055] | -0.003  [-0.022, 0.016] | - |  |
| 1. Age | -0.042  [-0.054, -0.029] | 0.030  [0.018, 0.042] | 0.023  [0.011, 0.035] | 0.051  [0.032, 0.070] | - |
| 1. Ethnicity | 0.166  [0.154,0.178] | 0.035  [0.023, 0.047] | 0.004  [-0.009, 0.016] | 0.024  [0.012, 0.037] | -0.022  [-0.041, -0.002] |

**Figure S4. Distribution of neural synchrony and personality similarity in the EEG sample (n = 225)**


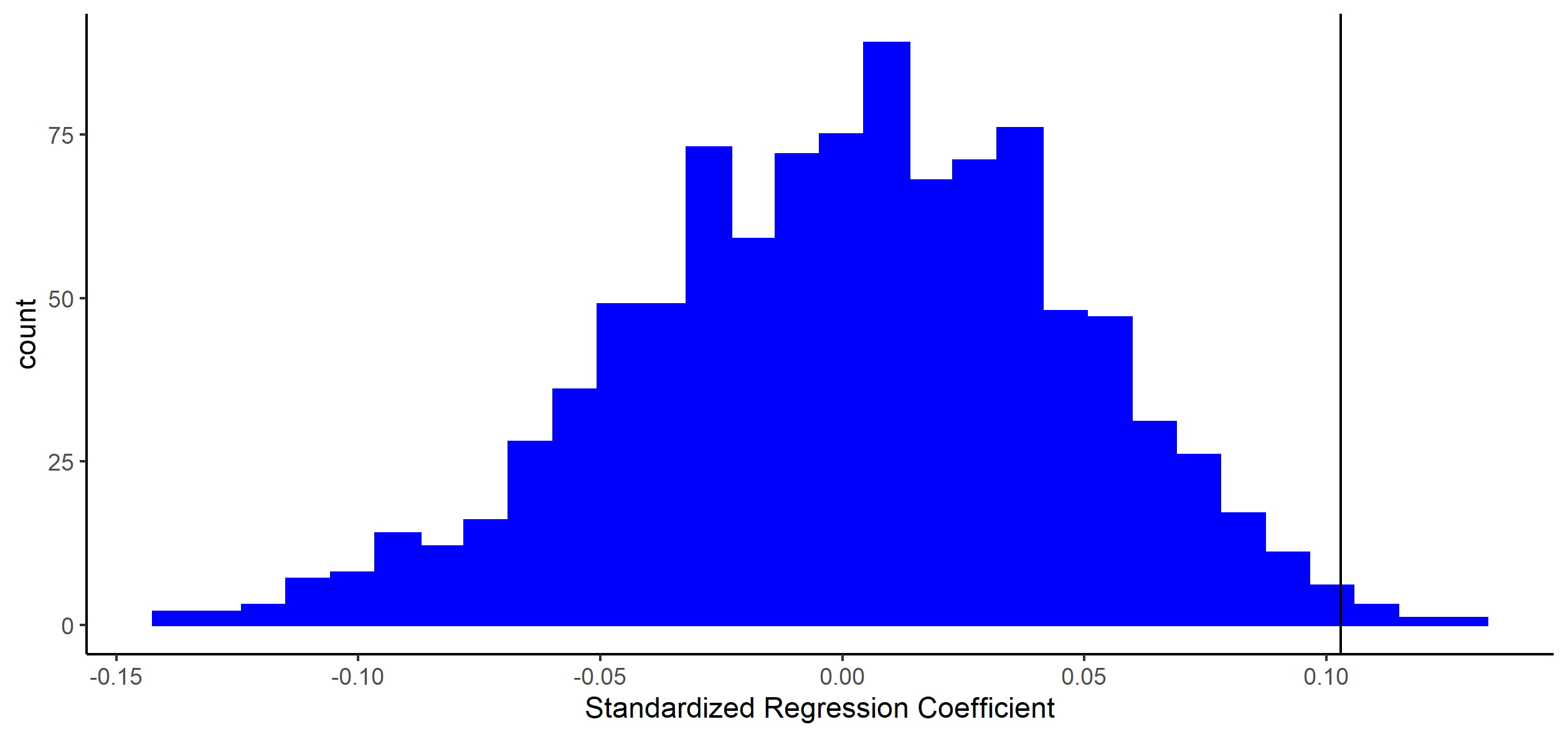


**Figure S4. Permutation testing histograms for the relationship between personality similarity and neural synchrony when controlling for socio-demographic variables.** For each statistical test, corresponding null distributions of sham beta coefficients for personality similarity were generated via permutation testing. The resulting null distributions are shown relative to the true beta coefficient for personality similarity (depicted by the vertical black line).

**References**

1. Esteban, O. *et al.* fMRIPrep: a robust preprocessing pipeline for functional MRI. *Nat. Methods* **16**, 111–116 (2019).

2. Pedregosa, F. *et al.* Scikit-learn: Machine learning in Python. *J. Mach. Learn. Res.* **12**, 2825–2830 (2011).

3. Norman, K. A., Polyn, S. M., Detre, G. J. & Haxby, J. V. Beyond mind-reading: multi-voxel pattern analysis of fMRI data. *Trends Cogn. Sci.* **10**, 424–430 (2006).

4. Haxby, J. V, Connolly, A. C. & Guntupalli, J. S. Decoding neural representational spaces using multivariate pattern analysis. *Annu. Rev. Neurosci.* **37**, 435–456 (2014).
